# Antibiotic Exposure Aggravates *Bacteroides*-Linked Uremic Toxicity in the Gut-Kidney Axis

**DOI:** 10.3389/fimmu.2022.737536

**Published:** 2022-03-24

**Authors:** Navin Ray, Hoyoung Jeong, Dasom Kwon, Juil Kim, Yuseok Moon

**Affiliations:** ^1^ Laboratory of Mucosal Exposome and Biomodulation, Department of Integrative Biomedical Sciences, Pusan National University, Yangsan, South Korea; ^2^ Graduate Program of Genomic Data Sciences, Pusan National University, Yangsan, South Korea

**Keywords:** antibiotic exposure, dysbiotic microbiota, Bacteroides, gut barrier, chemotherapy, renointestinal distress

## Abstract

Epidemiological and experimental evidence has implicated a potent link between antibiotic exposure and susceptibility to various diseases. Clinically, antibiotic treatment during platinum chemotherapy is associated with poor prognosis in patients with malignancy. In the present study, mucosal antibiotic exposure was assessed for its impact on renal distress as a sequela of platinum-based chemotherapy. Clinical transcriptome dataset-based evaluations demonstrated that levels of dysbiosis-responsive genes were elevated during renal distress, indicating pathological communications between gut and kidney. Experimentally, mucosal exposure to streptomycin aggravated platinum-induced renal tubular lesions in a mouse model. Moreover, antibiotic-induced dysbiosis increased susceptibility to gut mucosal inflammation, epithelial disruption, and bacterial exposure in response to cisplatin treatment. Further investigation of the luminal microbes indicated that antibiotic-induced dysbiosis promoted the dominance of *Bacteroides* species. Moreover, the functional assessment of dysbiotic microbiota predicted tryptophan metabolic pathways. In particular, dysbiosis-responsive *Bacteroides acidifaciens* was associated with the production of the uremic toxin indoxyl sulfate and renal injuries. The results of this study including bacterial community-based evaluations provide new predictive insights into the interorgan communications and interventions against dysbiosis-associated disorders.

## Introduction

The human population has been exposed to anti-infective agents *via* dietary exposure and therapeutic application, leading to dysbiosis in the mucosa of organs and potently affecting human disease progression. Extensive investigations have suggested epidemiological associations between exposure to anti-infective agents and the risk of inflammatory disorders such as inflammatory bowel diseases and allergic diseases (1–3). In agreement with the epidemiological association, experimental evidence has suggested that antibiotic-induced alterations in the gut microbial community facilitate pro-inflammatory responses (4–6). During antibiotic therapy, loss of competitive beneficial commensals leads to the outgrowth of specific groups of opportunistic bacteria including *Clostridium difficile* and antibiotic-resistant *Enterococcus faecium*, which are important causative agents of hospital-acquired infections (4, 7). In addition to its effects on opportunistic infections, antibiotic treatment increases the translocation of indigenous bacteria across the colonic epithelium as a result of alterations in the intestinal microflora (5). It is mechanistically dependent upon the presence of goblet cells, and the formation of goblet cell-associated antigen passages in the colon may account for the increased inflammatory outcomes in immunocompromised hosts. Moreover, streptomycin-induced inflammation can attenuate resistance to colonization by facultative anaerobic bacteria, such as *Escherichia coli*, in the gut (6). Intestinal exposure to streptomycin induces nitric oxide synthase 2 in the recruited inflammatory cells, which provides advantages of growth and competition for nitrate-respiring *E. coli* (6). Despite these observations of altered bacterial communities and several pathological events caused by antibiotics, the mechanism by which gastrointestinal exposure to antibiotic agents alters susceptibility to human diseases in other organs remains largely unknown.

Kidney is one of susceptible organs in response to the microbial dysbiosis. The gut–derived factors can account for mechanistic features of pathological processes in human renal diseases including acute kidney injury (AKI) and chronic kidney diseases (CKD) (8). Gut-derived bacterial endotoxin such as the hydrophobic anchor of lipopolysaccharide (LPS) which constitutes the outer membranes of most Gram-negative bacteria is primarily recognized and delivered by LPS-binding protein and CD14 in the circulation. Delivered LPS is further recognized by cellular toll like receptor 4 (TLR4) and myeloid differentiation factor 2 (MD2) which transduce the danger signals into the nuclei to modulate the pro-inflammatory gene expression, leading to diverse systemic and renal hemodynamic distresses (9, 10). Moreover, other bacterial components including peptidoglycans pose the endotoxin-like activities, showing synergistic actions with LPS to cause inflammatory insults in multiple organs (11). In addition to the bacterial components, gut bacteria-generated amino acid metabolites including phenols and indoles contribute to causing renovascular injuries (12, 13). In particular, circulating *p*-cresol sulfate and indoxyl sulfate are taken up or excreted through organic anion transporters in the kidney, but the accumulation of uremic toxins activates aryl hydrocarbon receptor (AhR), mediating oxidative and pro-inflammatory stress in patients with acute renal injuries or chronic kidney diseases (14–17). Reversely, uremic toxins induce the disruption of intestinal epithelial tight junction barriers, dysbiosis, and gut bacterial translocation, all of which aggravates the bacterial product-induced intestinal and renal inflammation and injuries (18).

Renal injury is a problematic clinical issue in patients receiving chemotherapy (19, 20). Although cisplatin is widely used in the treatment of solid tumors, such as head and neck, lung, bladder, testis, and ovarian cancers (21), patients receiving this cancer treatment may display complications such as AKI, one of the most common forms of renal disease (19). The general pathophysiological patterns of cisplatin-induced AKI include proximal tubular injury, oxidative stress, inflammation, and vascular injury in the kidney (21). A recent investigation suggested that antibiotic treatment during platinum chemotherapy is associated with decreased survival in patients with advanced epithelial ovarian cancer (22). In the present study, we assumed that antibiotic-induced dysbiosis mediates disease severity in extraintestinal tissues in patients under clinical treatment. The experimental model was designed to address impacts of dysbiosis on renal distress as a sequela of platinum-based chemotherapy. In addition to the pathological observations, the gut microbial community was evaluated as a key readout of the complex communication in gut-kidney axis. Ultimately, this study aimed to provide new insights into the personalized predictions of interorgan responses to gut microbial dysbiosis in clinical regimes including antibiotic treatment during chemotherapy.

## Materials and Methods

### Analysis Using Clinical Transcriptome-Based Datasets

Renal gene expression was assessed in patients with acute kidney injury (gse30718, n = 47) or chronic kidney disease (gse66494, n = 61). Since human kidney biopsies of patients with AKI are limited, patients with kidney explants were followed up as models of human AKI (GEO: gse30718) (23). Biopsies from transplants with AKI were compared with pristine protocol biopsies of stable transplants. Analyses of renal biopsies from patients with CKD were conducted to address the responsible genes associated with tubulointerstitial fibrosis and tubular cell injury trough two independent discovery and validation processes (24).

### Chemically-Induced Acute Renal Injury Model

Male C57BL/6 mice (6 weeks old, 16–18 g on average) were purchased from Jackson Laboratories (Bar Harbor, ME, USA). The mice were acclimated for 14 days prior to the experiments and maintained at 22 ± 2°C under 45–55% relative humidity and 12-h light/dark cycles. All mice of the same age were put in one cage and then were randomly transferred to different cages without any bias. Cages of mice were randomly assigned into groups belonging to a specific treatment group. The mice were housed three per cage and provided with sufficient food and water in environmentally protected cages comprising a transparent polypropylene body and a stainless-steel wire top cover. All animal care and experimental procedures were conducted in accordance with the guidelines of the Institutional Animal Care and Use Committee. This animal study was approved by the Pusan National University Institutional Animal Care and Use Committee (PNU-IACUC) (PNU-2015-0786). Eight-week-old mice (22–25 g) were pretreated with streptomycin (20 mg/mouse, Sigma-Aldrich, St. Louis, MO, USA) or *Bacteroide acidifaciens via* oral gavage. After 24 h of the antibiotic treatment, acute kidney injury was induced by an intraperitoneal injection of cisplatin (20 mg/kg). Mice were sacrificed after 72 hours of cisplatin treatment to analyze tissues. All animals were sacrificed under deep ether anesthesia. To address the effects of *Bacteroide acidifaciens* on the chemical-induced distress, C57BL/6 were daily administered with 5x10^9^ cfu/mL of *Bacteroide acidifaciens* in phosphate-buffered saline *via* oral gavage for 4 days. On the third day of microbial exposure, acute kidney injury was induced by an intraperitoneal injection of cisplatin (20 mg/kg) for 72 hours as mentioned.

### Histopathology

The kidney sections were stained with Periodic acid-Schiff (PAS) and microphotographs of the sections were taken using inverted microscope (Nikon-Eclipse Ts2R, Tokyo, Japan). The obtained images were quantified using adobe photoshop CS6 and multi Gauge V3.0 software. For the gut analysis, the small intestine was immediately removed, fixed in Carnoy’s solution, and embedded in paraffin. Sections were dewaxed using xylene, rehydrated using a series of graded alcohol solutions, and stained with hematoxylin and eosin (H&E) using a laboratory protocol to reveal the histopathological lesions. One of the kidneys was fixed with 4% PFA at 4°C, embedded in paraffin wax, and then stained with periodic acid-Schiff. The other part of the kidney was cut into two parts for RNA and protein isolation and snap-frozen in liquid nitrogen and homogenized on the day of organ harvest. The villous and crypt lengths of the jejunum and ileum were measured using a microscopic imaging software (NIS-Elements, Nikon Instruments, Tokyo, Japan). At least nine villi from each section were measured and averaged for each group. The morphological evaluation was performed using well-established criteria in a blinded manner (25). In brief, H&E‐stained cross‐sections of the small intestinal tissue were scored on a 0–4 scale for severity of histopathology based on the following criteria: 0, no change from normal tissue; Grade 1, mild inflammation present in the mucosa, comprised mainly of mononuclear cells, with little epithelial damage; Grade 2, multifocal inflammation greater than a Grade 1 score, with the observation of mononuclear and few polymorphonuclear cells (neutrophils), crypt glands pulled away from the basement membrane, mucin depletion from goblet cells, and the epithelium occasionally pulled away from the mucosa into the lumen; Grade 3, mutlifocal inflammation greater than a Grade 2 score, including mononuclear cells and neutrophils progressing into the submucosa, crypt abscesses present with increased mucin depletion, and presence of epithelial disruption; Grade 4, crypts no longer present, severe mucosal inflammation mainly composed of neutrophils, and epithelium no longer present or completely detached. An average of three fields of view per small intestine was determined for each mouse.

### Alcian Blue Staining

The prepared tissue sections were deparaffinized in xylene for 10 min, rehydrated in ethanol (100%, 90%, 80%, 70%, and 50%) for 3 min each, and then placed in distilled water for 10 min. AB 8GX (1% [w/v] alcian blue 8GX, Biosesang, Seoul, Korea) solution was applied to the sections for 30 min at room temperature, followed by a 2 min wash under running tap water. Counterstaining was performed for 5 min with 0.1% (w/v) Nuclear Fast Red, followed by washing for 2 min under running tap water. Stained sections were then dehydrated in two changes of 95% ethanol and two changes in absolute alcohol. The dried sections were cleared using xylene for a few minutes and then mounted with a synthetic mountant (Thermo Fisher Scientific, Seoul, Korea).

### Serum Creatinine and Urea Nitrogen Levels

Creatinine was measured using the Creatinine Serum Detection Kit (KB02-H2, Arbor Assays, Ann Arbor, MI, USA) according to the manufacturer’s instructions. Briefly, the mice were anesthetized with 30 µL of isoflurane (Hana Pharm Co., Seoul, Korea) and blood was collected in 1.5 mL tube containing 10 μL of 0.5 M EDTA by performing a retro-orbital sinus puncture and centrifuged at 1000 × g for 15 min to separate the plasma, followed by storage at −80°C. Before performing the assay, all samples were centrifuged at 18,341 × g for 15 min. Then, standard solutions (25 μL) with known concentrations of creatinine stock, blood samples, or water (blank), diluted with 25 μL of assay diluent, were mixed with 100 μL of DetectX Creatinine Reagent in a 96-well microplate and incubated for 30 min. Optical density was measured using a microplate reader at 490 nm wavelength. BUN (Blood Urea Nitrogen) was measured using a urea nitrogen colorimetric detection kit (KB024-H1, Arbor Assays), according to the manufacturer’s instructions. Briefly, plasma was centrifuged at 18,341 × g for 10 min and diluted in distilled water (1:20). Fifty microliters of the samples or appropriate standards were pipetted into a 96-well plate in duplicate. Next, 75 µL of color reagents A and B were added to each well using a repeater pipette. The plate was incubated at 25°C for 30 min, and the optical density was measured at 450 nm.

### Measurement of Indoxyl Sulfate

Quantitative analysis of the indole metabolite was performed using high performance liquid chromatography (HPLC) system with the autosampler and the fluorescence detector (RF-10Axl module) (Shimadzu, Kyoto, Japan). Three μL of the blood plasma diluted in 27 μL of 10 mM sodium acetate buffer (pH 4.5). Five μL of the diluted plasma sample was mixed with 36 μL of 10 ng/ml methyl-paraben, and the supernatants were collected after centrifugation at 10,000 × *g* for 5 minutes. Twenty μL of the supernatant was run in a C18 LC column in the mobile phase [10 mM sodium acetate buffer (pH 4.5) plus acetonitrile (10:90, v/v)] at 40°C for 20 minutes at the flow rate of 1 mL/min. The excitation and emission wavelengths of the detector were set at 280nm and 375nm, respectively.

### Conventional and Real-Time Reverse Transcription-Polymerase Chain Reaction

Frozen tissues were homogenized in 1 mL RiboEx solution (GeneAll Biotechnology, Seoul, Korea) containing stainless steel beads (5 mm) for 3−6 min using TissueLyser II (Qiagen). Total RNA was extracted using RiboEx (GeneAll Biotech, Seoul, South Korea) according to the manufacturer’s instructions. Then, RNA (500 ng) from each sample was transcribed to cDNA using a TOPscript RT DryMIX kit cDNA synthesis kit (Enzynomics, Daejeon, Korea). The cDNA was amplified using N-Taq DNA polymerase (Enzynomics) in a MyCycler Thermal Cycler (Bio-Rad) using the following parameters: denaturation at 95°C for 5 min, followed by 25 cycles of denaturation at 95°C for 10 s, annealing at 60°C for 15 s, and elongation at 72°C for 30 s. An aliquot of each polymerase chain reaction (PCR) product was subjected to 1.2% (w/v) agarose gel electrophoresis and visualized by staining with ethidium bromide. For real-time PCR, cDNA was amplified using SYBR green (SG, TOPreal™ qPCR 2X PreMIX, Enzynomics), performed with Rotor-Gene Q (Qiagen, Hilden, Germany) using the following parameters: denaturation at 94°C for 2 min, followed by 40 cycles of denaturation at 98°C for 10 s, annealing at 59°C for 30 s, and elongation at 98°C for 45 s. Each sample was evaluated in triplicate to ensure statistical robustness. Relative quantification of gene expression was performed using the comparative Ct method, where the Ct value was defined as the point at which a statistically significant increase in fluorescence was observed. The number of PCR cycles (Ct) required for fluorescence intensities to exceed a threshold value just above the background level was calculated for the test and reference reactions. GAPDH was used as an internal control for all the experiments.

### Stool Sample Collection and DNA Extraction

After 72 hours of CP administration, stool samples (5-6 pieces) (100 mg) of fecal samples were collected before animal sacrifice. The collected stool samples were stored at -150°C prior to DNA extraction. Microbial DNA was extracted and purified from 100 mg of the fecal sample using Exgene Stool DNA mini kit (GeneALL, Seoul, Korea) according to the manufacturer’s instructions. The extracted DNA was quantified using Qubit fluorometer and a high-sensitivity dsDNA reagent kit (Invitrogen, Carlsbad, CA, USA).

### Amplification of the 16S rRNA Genes and Library Preparation

A total of 13 DNA samples were pooled as treatment groups for a 16S metagenomic library preparation. The V3–V4 region of the 16S rRNA genes was PCR-amplified with a primer set (341F, 5´-CCTACGGGNGGCWGCAG-3´; 805R, 5´-GACTACHVGGGTATCTAATCC-3´) and Illumina sequencing adaptors (Illumina Inc.) using the KAPA HiFi HotStart Ready Mix (KAPA Biosystems, Wilmington, WA, USA) under the following cycling conditions: initial denaturation 95°C for 3 min, followed by 25 cycles of denaturation at 95°C for 30 s, annealing at 55°C for 30 s, extension at 72°C for 30 s, and a final extension at 72°C for 5 min. After amplicon purification using AMPure^®^ XP beads (Agencourt Biosciences, Beverly, MA), the PCR products were verified for library size using a BioAnalyzer (Agilent Technologies, Palo Alto, CA, USA), and the quantity was measured using a Qubit fluorometer. Then, the PCR amplicon was subjected to indexing PCR using the Nextera XT Index Kit (Illumina, Inc.). The PCR cycling conditions were as follows: 95°C for 3 min, followed by 8 cycles of denaturation at 95°C for 30 s, annealing at 55°C for 30 s, extension at 72°C for 30 s, and a final extension at 72°C for 5 min. The indexed PCR amplicons were purified using AMPure^®^ XP beads, verified for size using a BioAnalyzer, and quantified using the Qubit fluorometer. The quantified amplicons were diluted to 4 nM and pooled together for sequencing on an Illumina MiSeq platform (Illumina, Inc.), targeting 2× 300 bp paired-end sequence reads.

### Microbiome Data Processing

All sequences were quality-filtered and the primers were trimmed using Trimmomatic (v0.39) (26) with the following parameters: LEADING:3 TRAILING:3 MINLEN:36 SLIDINGWINDOW:4:15. The read pairs passing the quality filter were further analyzed using the Quantitative Insights Into Microbial Ecology 2 (QIIME2, v2020.2) pipeline (27). Using the DADA2 algorithm (28), the 34 bases of the reverse reads were truncated for quality improvement. The read pairs were then joined, denoised, and dereplicated, and chimeras were removed. The detailed data were used to call ASVs. The 16S rRNA representative sequences were assigned to taxonomic groups using the QIIME2 naive Bayes classifier trained on 99% operational taxonomic units (OTUs) and the primer region from the SILVA rRNA database (v138) (29). The taxonomic classification was visualized using the QIIME2 taxon barplot plugin. The 16S rRNA representative sequences were subjected to masked multisequence alignment using MAFFT (30, 31). A phylogenetic tree was constructed using FastTree (32). A heatmap, depicting the percentage abundance of OTUs, the relative abundance of which was in the top 30 in any sample, was generated using the R package, QIIME2R (v0.99.22). A phylogenetic tree based on the abundant OTUs was constructed and visualized using the neighbor-joining algorithm with Jukes-Cantor correction using MEGA X (33). Additionally, we used the CLcommunity™ software for analysis. PICRUSt2 (Phylogenetic Investigation of Communities by Reconstruction of Unobserved States) was used as a software for predicting functional abundances based only on marker gene sequences. PICRUSt2 (Phylogenetic Investigation of Communities by Reconstruction of Unobserved States) was used as a software for the functional differences between different groups. It is based on 16S rRNA sequencing data annotated by the Greengenes database for predicting functional composition of known microbial genes.

### Indole Production Test

*Bacteroide acidifaciens* was cultured in the Gifu anaerobic media (GAM) for 48 hours. Additionally, the bacteria were further cultured in the minimal media with or without tryptophan (5mg/ml) for 6 hours. Two mL of each culture media (GAM or the minimal media) was mixed with 500 uL laboratory grade xylene. The mixture was allowed to settle for 5 minutes and was slowly added with 5-10 drops of the Ehrlich’s reagent to detect the presence of pink-colored indole produced by the bacteria.

### Statistical Analysis and Reproducibility

Statistical analysis was performed using GraphPad Prism 6 software (GraphPad Software, La Jolla, CA, USA). *Student*’s t-test was used for comparative analysis of the two groups of data. To compare multiple groups, the data were subjected to analysis of variance (ANOVA) with *Newman–Keuls* method as a *post hoc* ANOVA assessment. For two gene correlation coefficient (R) determination in clinical datasets, *Pearson*’s correlation analysis was performed. All evaluations are representative of two or three independent experiments. Details of the number of biological replicates and the assays are given in each figure legend.

## Results

### Patients With Renal Distress Display an Increased Expression of Gut Injury Biomarkers

**Figure 1:**
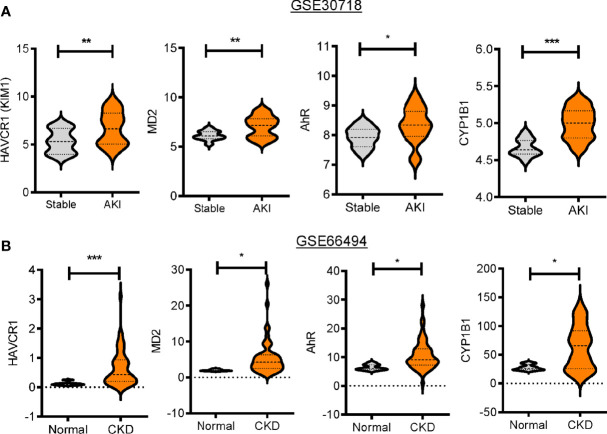
Expression profiles of bacterial dysbiosis-responsive biomarker genes in patients with renal distress. Expression of bacterial dysbiosis-responsive biomarker genes was assessed in patients with acute kidney injury (gse30718, n = 47, **A**) or chronic kidney disease (gse66494, n = 61, **B**). Results are shown in the violin plot as mean values ± SD and the asterisks (∗) indicate significant differences between two groups using *Student*’s t-test (*p < 0.05, **p < 0.01, ***p < 0.001).

Based on an assumption that dysbiosis-linked stress may affect renal diseases, clinical dataset analyses were performed to determine the bacterial dysbiosis-responsive molecules in patients with kidney diseases, including AKI and chronic kidney disease (CKD). First, patients with AKI and CKD displayed increased levels of renal injury molecule 1 (*KIM-1*, also known as *HAVCR1*) (**Figure 1**). Since renal cells may be exposed to circulating endotoxins and uremic toxins during dysbiosis, we evaluated the bacterial ligand-responsive receptor genes in clinical transcriptome. Whereas the levels of *LPS-binding protein, CD14*, and *TLR4* were marginally altered (data not shown), the AKI group displayed significantly higher levels of *MD2* than the pristine biopsy control group (**Figure 1A**). Moreover, patients with CKD had increased levels of *MD2* compared to healthy controls (**Figure 1B**). In addition to *MD2*, the expressions of *AhR* and its target gene *CYP1b1* were elevated in patients with renal distress (**Figures 1A, B**). All of these results suggest that patients with acute and chronic renal disorders are potently exposed to dysbiosis-linked stressors, which will be verified in the chemical-induced acute renal injury model.

### Mucosal Streptomycin Exposure Aggravates Cisplatin-Induced Renal Injuries in Mice

**Figure 2:**
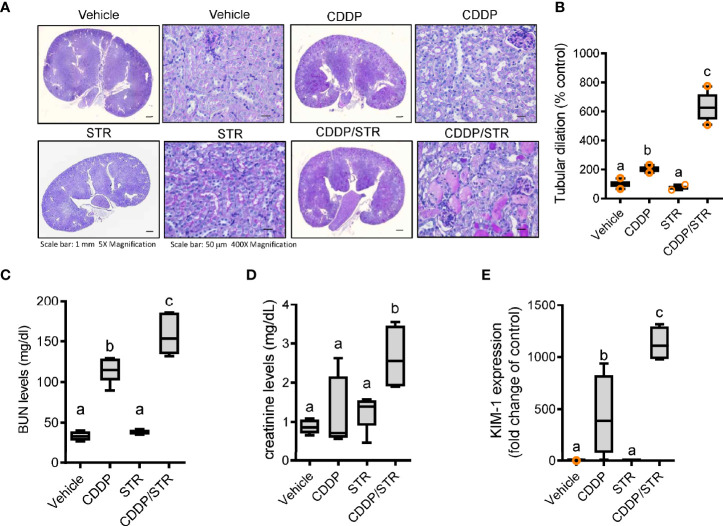
Effects of STR exposure on CDDP-induced renal injury. Eight-week-old wild type male mice (n = 3–5) were orally pre-exposed to the vehicle or streptomycin (20 mg/mouse) for 24 h and then renal injury was induced with CDDP (20 mg/kg) *via* intraperitoneal administration for an additional 72 h. **(A)** Periodic acid-Schiff (PAS) staining. **(B)** Tubular dilation levels according to PAS staining. **(C, D)** levels of blood urea nitrogen (BUN, **C**) and creatinine **(D)**. **(E)** The mRNA expression was measured in the mouse kidney using real-time RT-PCR. Results are representative of three independent experiments. The results are shown as a box plot with Tukey whiskers and the different letters (a–c) over each box represent significant differences between groups using ANOVA with Newman–Keuls method as a *post hoc* ANOVA assessment (p < 0.05).

On an assumption that gut dysbiosis is associated with renal diseases, we evaluated the effect of streptomycin exposure on cisplatin (CDDP)-induced renal injury. Microscopic gross observation of renal tissues confirmed the known histological outcomes of CDDP-induced renal injury, such as tubular dilation and loss (**Figure 2A**). Quantitative scoring of tubular damage revealed that CDDP treatment resulted in the formation of proximal tubule dilatation, vacuolization, and degeneration, which was aggravated in streptomycin (STR)-exposed subjects. In particular, STR-exposed mice displayed severe renal pathological outcomes in response to platinum therapy, such as prevalent cyst formation in both the cortex and outer medulla compared to those with no antibiotic exposure (**Figure 2B**). Moreover, quantitative evaluations of the humoral biomarkers of renal injuries, such as blood urea nitrogen (BUN) and creatinine, demonstrated that antibiotic exposure markedly increased CDDP-induced damage in mice (**Figures 2C, D**). In addition, renal mRNA expression of kidney injury molecule (KIM-1), a representative early biomarker of acute kidney injury, was elevated in mice with CDDP-induced AKI, and was superinduced by STR exposure, consistent with the severe histological renal injuries (**Figure 2E**).

### Streptomycin Increased Susceptibility to Intestinal Inflammation and Injuries in Mice

**Figure 3:**
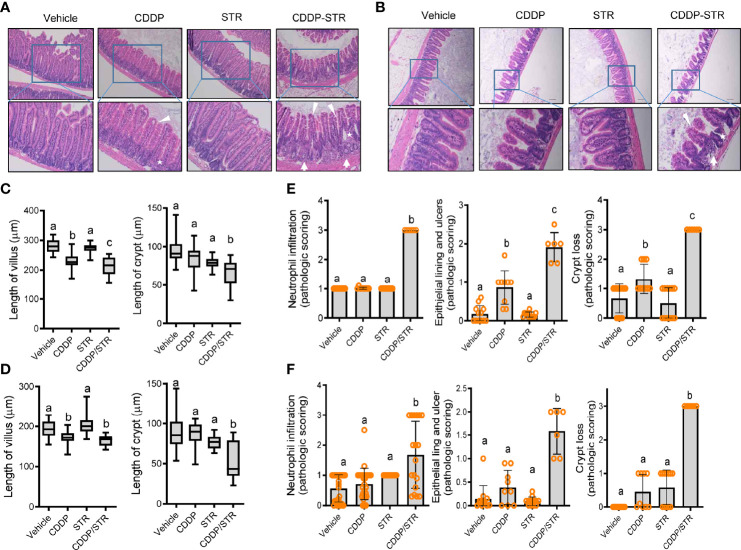
Histopathological effects of STR exposure on jejunum and ileum in CDDP-induced renal injury model. Eight-week-old wild type male mice (n = 3–5) were orally preexposed to the vehicle or streptomycin (20 mg/mouse) for 24 h and then renal injury was induced with CDDP (20 mg/kg) *via* intraperitoneal administration for an additional 72 h. **(A, B)** Histological observation of hematoxylin and eosin (H&E)-stained sections of the small intestine [jejunum **(A)** and ileum **(B)**] [magnification 100× (upper) and 200× (lower) with scale bar(s): 100 μm]. The arrows indicate neutrophil infiltration, stars indicate crypts, and arrowheads indicate epithelial lining integrity. **(C, D)** Quantitative comparisons of villus length (left) and crypt length (right). The results are shown as a box plot with Tukey whiskers and the different letters (a–c) over each box represent significant differences between groups (p < 0.05) in the jejunum **(C)** and the ileum **(D)**. **(E, F)** Quantitative pathological scoring of neutrophilic infiltration (left), epithelial lining and ulcers (middle), and crypt loss (right) in the jejunum **(E)** and the ileum **(F)**. Results are representative of three independent experiments. The results are shown as a bar graph and the different letters over each bar represent significant differences between the groups using ANOVA with Newman–Keuls method as a *post hoc* ANOVA assessment (p < 0.05).

**Figure 4:**
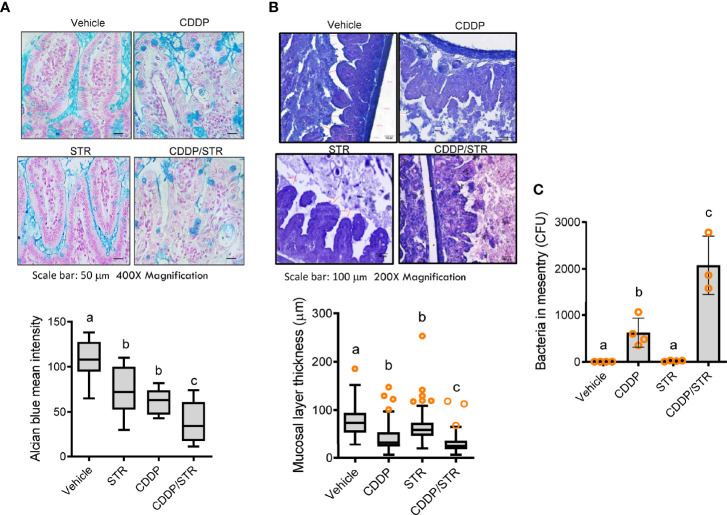
Effects of STR exposure on the intestinal mucosal barrier in CDDP-induced renal injury model. Eight-week-old wild type male mice (n = 3–5) were orally preexposed to the vehicle or streptomycin (20 mg/mouse) for 24 h and then renal injury was induced with CDDP (20 mg/kg) *via* intraperitoneal administration for an additional 72 h. Results are representative of three independent experiments. **(A)** Secreted and intracellular mucins were stained with Alcian blue (blue colored, magnification 400×, scale bar: 100 μm). Relative levels of the secreted mucin were measured (the lower graph). The results are shown as a plot with Tukey whiskers and the different letters (a–c) over each box represent significant differences between groups (p < 0.05). **(B)** Representative images of the intestinal mucosa with Gram staining [magnification 200×, scale bar(s) 100 μm]. The mucosal layer thickness was measured (the lower graph). The results are shown as a plot with Tukey whiskers and the different letters (a–c) over each box represent significant differences between groups (p < 0.05). **(C)** The bacteria detected in the mesenteric lymph node (MLN) were quantified. The different letters represent significant differences between groups using ANOVA with Newman–Keuls method as a *post hoc* ANOVA assessment (p < 0.05).

Based on clinical transcriptome-based evaluation, renal distress was assumed to be associated with gut-derived factors. Therefore, we assessed the impact of antibiotic exposure on gut integrity. Morphologically, both the crypts and villi of the STR-exposed intestine were significantly shortened by CDDP treatment while only CDDP-exposed tissues displayed shortened crypts (**Figures 3A**–**D**). Moreover, histopathological evaluations demonstrated that STR exposure aggravated CDDP-induced pathological distress, which comprised severe neutrophil infiltration, epithelial lining destruction, and crypt loss (**Figures 3E, F**). The gut epithelial lining contains goblet cells that secrete mucus to establish a protective mucosal layer between the luminal environment and the epithelial layer. CDDP treatment attenuated mucus production in the gut, which was remarkable in the STR-exposed mice (**Figure 4A**). Alcian blue-based staining and quantitation showed severe deficiency in mucin-producing goblet cells of the villus in mice co-exposed to STR and CDDP. Moreover, the total thickness of the mucosal layer was similarly affected. Gram staining was performed to localize luminal bacteria and measure the thickness of the inner mucosal layer. The STR-exposed epithelial lining closely faces the luminal matter containing dietary and microbial factors due to the reduced mucosal barrier (**Figure 4B**). Subsequently, luminal bacteria can easily translocate into the inner tissues under a disrupted gut barrier. CDDP treatment enhanced the translocation of the luminal bacteria into the mesenteric lymph nodes, which was notably increased in STR-exposed mice (**Figure 4C**). Taken in sum, antibiotic exposure aggravated CDDP-induced gut barrier integrity.

### Gut Bacterial Diversity and Composition in Response to Streptomycin Exposure

**Figure 5:**
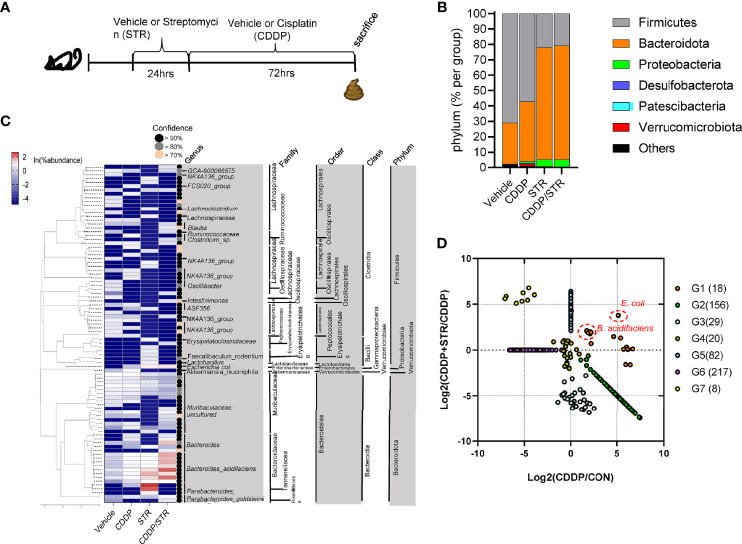
Effects of STR exposure on the murine gut microbiota profile in CDDP-induced renal injury model. **(A)** Eight-week-old wild type male mice (n = 3–5) were orally preexposed to the vehicle or streptomycin (20 mg/mouse) for 24 h and then renal injury was induced with CDDP (20 mg/kg) *via* intraperitoneal administration for an additional 72 h. **(B)** The fecal bacteria were subjected to 16S rRNA analysis for phylogenetic composition at the phylum level (B. the mean relative abundances of phyla). **(C)** The bacteria of each top 30 abundant taxa are listed along with the corresponding mean relative abundance. **(D)** Relative abundance and clustering of bacterial consortia in response to STR in CDDP-induced renal injury. Results are representative of two independent experiments.

In addition to changes in the mucosal barrier and luminal bacterial translocation, the luminal bacterial composition was evaluated as a potent etiology of aggravated renal distress in response to STR as schemed in the **Figure 5A**. A total of 1,239,235 paired-end reads were produced from the three libraries using the Illumina MiSeq platform (Illumina Inc., San Diego, CA, USA). After quality trimming and removing singletons using Trimmomatic (v0.39), 1,233,393 paired-end reads were subjected to further analyses using the QIIME2 pipeline. Using the DADA2 algorithm, 530 representative sequences of the V3–V4 region of 16S rRNA genes with an average length of 447.05 bp across three libraries were constructed, against which 12545, 11913, 7366, and 11223 features were counted in the vehicle, CDDP, STR, and CSSP+STR, respectively. The composition differed in the samples as Firmicutes contributed 71.5% and 57.2% of the total features as the most abundant phylum in the vehicle and CDDP groups, respectively, while Bacteroidota was the most abundant phylum in the antibiotic-exposed groups (**Figure 5B**). Moreover, the ratio of Proteobacteria was 13 times its original value in response to STR exposure. Closer observation of the communities at the family level demonstrated that Lachnospiraceae, as the major family, contributed approximately 33% in the vehicle and CDDP-exposed communities, respectively (**Supplementary Figure 1A**). However, Bacteroidaceae was the main family in the STR and CDDP-STR groups. In particular, the STR-exposed group showed a remarkable increase in the abundance of Bacteroidaceae (59.5%) in response to platinum treatment (**Supplementary Figure 1A**). The microbial community profile exhibited the top 30 most abundant amplicon sequence variants (ASVs) in each sample, in which the ASVs were clustered based on the similarity of their phylogenetic alignments. Whereas the relative abundance of the microbial community in the vehicle group was evenly distributed, the relative abundance distribution was skewed toward ASVs classified as *Bacteroides acidifaciens*; 13.36%, 29.08%, and 50.54% of the total features in CDDP, STR, and CDDP-STR, respectively, were classified as *Bacteroides acidifaciens* (**Figure 5C**). In addition, a relatively high abundance (7.46%) observed in the CDDP-STR group was also assigned to unclassified *Bacteroides*, which are closely clustered with *B. acidifaciens.* Along with *Bacteroides*, the abundance of *E. coli* increased by 3.99% and 4.61% in STR and CDDP-STR groups, respectively. Although the effect of CDDP treatment on the microbial community was too trivial to associate with disease outcomes, antibiotic exposure caused pronounced changes in gut microbiota composition, such as an increased proportion of Bacteroidetes and Proteobacteria, with a dramatic decrease in Firmicutes. In particular, *B. acidifaciens* and *E. coli* belonged to CDDP-specific and CDDP-STR-specific consortia (**Figure 5D**). This bi-specific pattern was also verified in the classification of bacteria based on ontological analysis using the Venn diagram (**Supplementary Figure 1B**). *Bacteroides acidifaciens* and *E. coli* shared consortia between the CDDP and CDDP-STR groups.

### Species-Level Analysis Based on Genes Involved in Renal Toxic Metabolite Production

**Figure 6:**
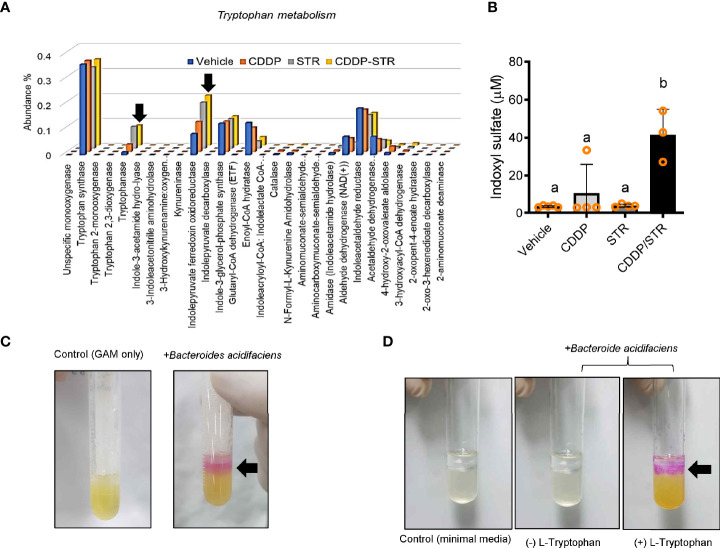
Effects of STR exposure on the bacterial tryptophan metabolism in CDDP-induced renal injury model. Eight-week-old wild type male mice (n = 3–5) were orally preexposed to the vehicle or streptomycin (20 mg/mouse) for 24 h and then renal injury was induced with CDDP (20 mg/kg) *via* intraperitoneal administration for an additional 72 h. The fecal bacteria were subjected to 16S rRNA analysis for the determination of the phylogenetic composition. **(A)** Genes related to tryptophan metabolism reconstructed from 16S rRNA profile-based metagenomic prediction using *PICRUSt2*. **(B)** The blood indoxyl sulfate levels were measured using high-performance liquid chromatography. The different letters represent significant differences between groups using ANOVA with Newman–Keuls method as a *post hoc* ANOVA assessment (p < 0.05). **(C, D)** Ehrlich’s test for indole production from *Bacteroides acidifaciens*. The bacteria were cultured in the Gifu anaerobic media (GAM) broth **(C)**, or minimal media with or without tryptophan (5mg/ml) **(D)**. Results are representative of three independent experiments.

**Figure 7:**
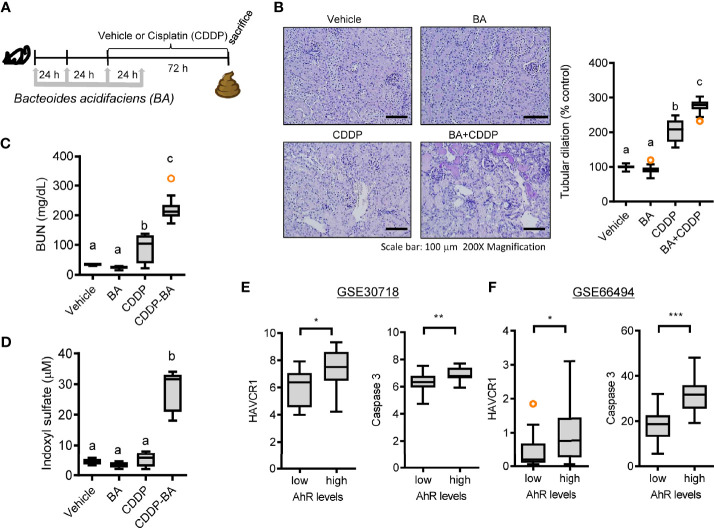
Effects of STR exposure on IS production and disease outcomes during renal injury. **(A)** Eight-week-old wild type male mice (n = 3–5) were daily preexposed to the vehicle or 5x10^9^ cfu/mL of *Bacteroide acidifaciens* in phosphate-buffered saline *via* oral gavage for 4 days. On the third day of microbial exposure, renal injury was induced with CDDP (20 mg/kg) *via* intraperitoneal administration for an additional 72 h. **(B)** PAS staining (the left panel) and tubular dilation levels according to PAS staining (the right graph). **(C)** levels of blood urea nitrogen (BUN). **(D)** The blood indoxyl sulfate levels were measured using high-performance liquid chromatography. Results are representative of two independent experiments. The results are shown as a plot with Tukey whiskers and the different letters (a–c) over each box represent significant differences between groups using ANOVA with Newman–Keuls method as a *post hoc* ANOVA assessment (p < 0.05). **(E, F)** Expression of renal injury biomarker genes was assessed in patients with acute kidney injury (gse30718, n = 47, **E**) or chronic kidney disease (gse66494, n = 61, **F**). Results are shown in a box plot with Tukey whiskers and the asterisks (∗) indicate significant differences between two groups using *Student*’s t-test (*p < 0.05, **p < 0.01, ***p < 0.001).

The bacterial community was evaluated for its genetic features of the metabolic enzymes involved in functionality. Changes in the microbiota composition and community structure are associated with the production of microbiota-derived uremic retention solutes (URS) (34, 35). The 16S rRNA profile-based functional prediction using *PICRUSt2* demonstrated indole-metabolic enzymes such as tryptophanase and indolepyruvate ferredoxin oxidoreductase were notably elevated in the microbiota exposed to STR (**Figure 6A**). Indole metabolites are a pivotal type of URS that accumulates in the blood and tissues during acute or chronic renal diseases through human and microbial tryptophan metabolism. We addressed bacteria with genes encoding URS-producing enzymes in the present bacterial community. Based on sequence alignments of the bacterial genes, ten different species of bacteria were identified as having genes for tryptophanase (TNase) (*B. acidifaciens, E. coli, Akkermansia muciniphila, B. uniformis, B. xylanisolvens, Alistipes finegoldii, Oscillibacter, Anaerotruncus colihominis, Porphyromonadaceae*, and *B. faecis*), aryl sulfotransferase (*Bifidobacterium longum* group and *Lactobacillus plantarum*), and choline TMA-lyase (CutC) (*E. coli*). In particular, bacterial TNase is the key enzyme for the conversion of tryptophan into indole in the gastrointestinal tract, which is then further metabolized into renal toxic indoxyl sulfate (IS) in the liver by the hepatic CYP450 enzyme. Consistent with the microbial genetic features of indole metabolism, the blood level of IS was significantly elevated by CDDP treatment in STR-exposed animals whose microbiota showed an elevated composition of *B. acidifaciens* (**Figure 6B**). On an assumption that *B. acidifaciens* is a pivotal IS producer in renal injury model, this bacterium was assessed for its ability to convert tryptophan into indole *in vitro*. *B. acidifaciens* produced indole in GAM broth which already includes tryptophan (**Figure 6C**). Although the bacteria displayed Ehrlich’s test negative in the minimal media without tryptophan, they produced indole in the presence of tryptophan (**Figure 6D**). Next, we evaluated actions of *B. acidifaciens* in CDDP-induced renal injury model (**Figure 7A**). Pre-exposure to *B. acidifaciens* aggravated CDDP-induced renal injury although only the bacterial exposure had marginal effects on the pathological score and BUN levels (**Figures 7B, C**, respectively). Moreover, *B. acidifaciens*-pretreated animals showed notable elevations of plasma IS in response to CDDP (**Figure 7D**). As mentioned in the introduction, exposure to IS activates AhR-linked signaling which mediates oxidative and pro-inflammatory stress in patients with acute renal injuries or chronic kidney diseases (14–17). Mechanistically, it was assumed that AhR signaling is involved in stress responses in patients with renal distresses since AhR expression was elevated during renal injury (**Figure 1**). Subjects with high AhR expression presented high levels of renal injury molecule 1 (also known as *HAVCR1*) and the apoptosis marker caspase 3 (**Figures 7E, F**), suggesting a positive association between IS-responsive AhR signaling and renal distress.

## Discussion

Oral antibiotic exposure increased the susceptibility to acute renointestinal distress in the present study *via* potent changes in microbial community. In the present model, only STR-induced dysbiosis had marginal effects on acute pathological outcomes. In order to have detrimental effects on extraintestinal tissues, the harmful bacteria or their metabolites need to translocate the epithelial and vascular barrier in the gastrointestinal tract. CDDP treatment facilitated the microbial translocation (**Figure 4C**) by disrupting the epithelial lining (**Figures 3E, F**). Therefore, STR-induced dysbiosis exerted aggravating actions in the presence of CDDP potently *via* a disrupted gut barrier. Moreover, since uremic toxins and urea-derived metabolites can cause the degradation of tight junction proteins of the gut epithelial lining in CKD model (36, 37), it is speculated that the chronic dysbiosis-induced URS could lead to leaky gut and subsequent renal distress. URS-induced renal injuries mostly result from inflammatory responses and reactive oxygen species (ROS) production. Circulating *p*-cresol sulfate and indoxyl sulfate can activate AhR-linked signaling in renal cells, leading to oxidative and inflammatory stress during acute renal injuries or chronic kidney diseases (14–17). The present evaluations using clinical transcriptome also verified the positive association between AhR and renal injury biomarker genes in patients with AKI or CKD. Moreover, IS-producing *B. acidifaciens* was proven to elevate renal injury-indicating BUN levels in the experimental AKI model. Dysbiosis-induced URS, including IS, acts on the basolateral membrane of renal proximal tubular cells by binding to the organic anion transporter, and causes inflammation and nephrotoxicity *via* the generation of ROS, pro-inflammatory cytokines, hypoxia factors, and pro-fibrotic factors (38, 39). Approximately 190 gut microbial operational taxonomic units are altered and contribute to URS production in patients with end-stage renal disease (ESRD) (40, 41). A previous *in vitro* evaluation indicated that *B. thetaiotaomicron* and *B. ovatus* are the main producers of indole, whereas other *Bacteroides*, including *B. vulgatus, B. caccae*, and *B. fragilis* are nonproducers in the human gut (42). In the present study, instead of the main indole-producing *Bacteroides* species, such as *B. thetaiotaomicron* and *B. ovatus*, the dysbiosis-linked dominant *B. acidifaciens* was associated with IS-linked pathological outcomes in the mouse gut and kidney. Moreover, while *B. uniformis* and *B. xylanisolvens* marginally contributed to the TNase pool in the gut exposed to STR, the abundance of the non-*Bacteroides* genus *Escherichia* can account for the increased levels of total bacterial TNase expression in response to STR. In addition to URSs, circulating endotoxins from Gram-negative bacteria such as *E. coli* can exert detrimental actions in systemic and vascular inflammation in CKD (10). Since MD2, a receptor signaling molecule of endotoxin, was also elevated in human CKD, experimental and clinical evidence-based evaluations are warranted to address endotoxin-responsive renal injuries during dysbiosis. Moreover, since the profile of the present study using an animal model is different from that in the human niche, validation in patients with antibiotic treatment is necessary for better clinical application.

In contrast to the predictions and experimental evidence in the actions of indole-producing *B. acidifaciens* in renal pathological outcomes, the bacteria have been assumed beneficial in the murine models of metabolic diseases (43–45). The bacteria can improve glucose homeostasis by increasing the levels of serum insulin and glucagon-like peptide-1 in mice (43). Moreover, *B. acidifaciens* causes alterations in the bile acid profiles, which promotes fat oxidation and mitogenesis in the PPAR alpha signaling pathway. All these events may contribute to microbial intervention in metabolic diseases such as diabetes and obesity (43). In addition to its metabolic aspects, *B. acidifaciens* improves mucosal barrier integrity. In particular, *B. acidifaciens* increases the population of T cell-dependent IgA-positive B cells and the subsequent mucosal IgA secretion in the colon (44), which can retard pathogen colonization. Moreover, *B. acidifaciens-*produced propionic acid mediates colonization resistance to gut pathogens, including *Salmonella* and *Listeria* species (45). Moreover, soluble high-fiber diet-induced beneficial actions, including IgA production and radiosensitization, are positively correlated with *B. acidifaciens* abundance in the gut microbial community (46, 47). Therefore, similarly, the dysbiosis-linked abundance of *B. acidifaciens* can counteract the colonization of dysbiosis-linked pathogens and pathobionts during antibiotic treatment or other insults *via* the production of muco-protective factors, such as IgA and propionate. These opposite outcomes in different diseases need to be carefully addressed in terms of human relevance since there is no clinically proven report. Acute exposure to the bacteria aggravated renal distress as a sequela of platinum-based chemotherapy in the murine model. In this model, only the bacterial exposure had no obvious adverse outcomes in the kidney and gut. However, the bacterial actions were detrimental when the gut barrier integrity was damaged under the therapeutic treatment. Otherwise, mucosa-restricted actions of the bacteria would be beneficial as shown in previous reports (43–45). However, the results in the present model of platinum-mediated sequela implicate the bacterial risk under clinical regime. Antibiotic treatment during platinum-based chemotherapy is closely associated with decreased survival and recurrence in patients with advanced epithelial ovarian cancer (22). Therefore, considering the mucositis-linked gut distress during the chemotherapy, the gut bacteria such as *B. acidifaciens* would be exposed to the circulatory system and extraintestinal organs, acting differently in new circumstances. The present findings provide clinically relevant insights into the adverse outcomes of the bacterial exposure in patients under the chemotherapy or gastrointestinal insult.

Conclusively, the present study evaluated renointestinal distress in response to mucosal antibiotic exposure. Clinical transcriptomic evaluation in patients with acute or chronic kidney diseases indicated potent associations between dysbiosis and renal distress. Antibiotic exposure aggravated the CDDP-induced pathological events in gut and kidney. STR-exposed subjects displayed severe renointestinal injuries, including renal tubular and gut inflammation, compared to the antibiotic-free groups. In addition to the histopathological changes, populations of indole-producing bacteria including *B. acidifaciens* and some of the pathobionts increased during dysbiosis. In particular, *B. acidifaciens* contributed to uremic toxicity and renal injuries in response to dysbiosis. Taken together, risk evaluations of mucosal dysbiosis-linked host events in association with bacterial community provided crucial insights into disease severity or interventions against environmental insult in the gut-kidney axis.

## Data Availability Statement

The datasets presented in this study can be found in online repositories. The names of the repository/repositories and accession number(s) can be found below: SRA, SAMN20309880.

## Ethics Statement

All animal care and experimental procedures were conducted in accordance with the guidelines of the Institutional Animal Care and Use Committee. This animal study was approved by the Pusan National University Institutional Animal Care and Use Committee (PNU-IACUC) (PNU-2015-0786).

## Author Contributions

Project design and hypotheses were defined by YM. YM, NR, JK, DK, and HJ conducted experiments and analyzed the data. YM prepared the manuscript. YM supervised the overall project. All authors contributed to the article and approved the submitted version.

## Funding

This research was supported by the Basic Science Research Program through the National Research Foundation of Korea (NRF) funded by the Ministry of Education (2018R1D1A3B05041889).

## Conflict of Interest

The authors declare that the research was conducted in the absence of any commercial or financial relationships that could be construed as a potential conflict of interest.

## Publisher’s Note

All claims expressed in this article are solely those of the authors and do not necessarily represent those of their affiliated organizations, or those of the publisher, the editors and the reviewers. Any product that may be evaluated in this article, or claim that may be made by its manufacturer, is not guaranteed or endorsed by the publisher.

## References

[1] Metsala J Lundqvist A Virta LJ Kaila M Gissler M Virtanen SM. Mother's and Offspring's Use of Antibiotics and Infant Allergy to Cow's Milk. Epidemiology (2013) 24(2):303–9. doi: 10.1097/EDE.0b013e31827f520f 23348066

[2] Shaw SY Blanchard JF Bernstein CN. Association Between the Use of Antibiotics and New Diagnoses of Crohn's Disease and Ulcerative Colitis. Am J Gastroenterol (2011) 106(12):2133–42. doi: 10.1038/ajg.2011.304 21912437

[3] Ungaro R Bernstein CN Gearry R Hviid A Kolho KL Kronman MP. Antibiotics Associated With Increased Risk of New-Onset Crohn's Disease But Not Ulcerative Colitis: A Meta-Analysis. Am J Gastroenterol (2014) 109(11):1728–38. doi: 10.1038/ajg.2014.246 25223575

[4] Hendrickx AP Top J Bayjanov JR Kemperman H Rogers MR Paganelli FL. Antibiotic-Driven Dysbiosis Mediates Intraluminal Agglutination and Alternative Segregation of Enterococcus Faecium From the Intestinal Epithelium. mBio (2015) 6(6):e01346-15. doi: 10.1128/mBio.01346-15 26556272 PMC4659461

[5] Knoop KA McDonald KG Kulkarni DH Newberry RD. Antibiotics Promote Inflammation Through the Translocation of Native Commensal Colonic Bacteria. Gut (2016) 65(7):1100–9. doi: 10.1136/gutjnl-2014-309059 PMC4670297 26045138

[6] Spees AM Wangdi T Lopez CA Kingsbury DD Xavier MN Winter SE. Streptomycin-Induced Inflammation Enhances Escherichia Coli Gut Colonization Through Nitrate Respiration. mBio (2013) 4(4):e00430–13. doi: 10.1128/mBio.00430-13 PMC3705454 23820397

[7] Mullish BH Williams HR. Clostridium Difficile Infection and Antibiotic-Associated Diarrhoea. Clin Med (Lond) (2018) 18(3):237–41. doi: 10.7861/clinmedicine.18-3-237 PMC6334067 29858434

[8] Ramezani A Raj DS. The Gut Microbiome, Kidney Disease, and Targeted Interventions. J Am Soc Nephrol (2014) 25(4):657–70. doi: 10.1681/ASN.2013080905 PMC3968507 24231662

[9] Heyman SN Darmon D Goldfarb M Bitz H Shina A Rosen S. Endotoxin-Induced Renal Failure. I. A Role for Altered Renal Microcirculation. Exp Nephrol (2000) 8(4-5):266–74. doi: 10.1159/000020678 10940726

[10] McIntyre CW Harrison LE Eldehni MT Jefferies HJ Szeto CC John SG. Circulating Endotoxemia: A Novel Factor in Systemic Inflammation and Cardiovascular Disease in Chronic Kidney Disease. Clin J Am Soc Nephrol (2011) 6(1):133–41. doi: 10.2215/CJN.04610510 PMC3022234 20876680

[11] Wray GM Foster SJ Hinds CJ Thiemermann C. A Cell Wall Component From Pathogenic and non-Pathogenic Gram-Positive Bacteria (Peptidoglycan) Synergises With Endotoxin to Cause the Release of Tumour Necrosis Factor-Alpha, Nitric Oxide Production, Shock, and Multiple Organ Injury/Dysfunction in the Rat. Shock (2001) 15(2):135–42. doi: 10.1097/00024382-200115020-00010 11220642

[12] Barrios C Beaumont M Pallister T Villar J Goodrich JK Clark A. Gut-Microbiota-Metabolite Axis in Early Renal Function Decline. PloS One (2015) 10(8):e0134311. doi: 10.1371/journal.pone.0134311 26241311 PMC4524635

[13] van den Brand JA Mutsaers HA van Zuilen AD Blankestijn PJ van den Broek PH Russel FG. Uremic Solutes in Chronic Kidney Disease and Their Role in Progression. PloS One (2016) 11(12):e0168117. doi: 10.1371/journal.pone.0168117 28033375 PMC5199014

[14] Miyamoto Y Watanabe H Noguchi T Kotani S Nakajima M Kadowaki D. Organic Anion Transporters Play an Important Role in the Uptake of P-Cresyl Sulfate, a Uremic Toxin, in the Kidney. Nephrol Dial Transplant (2011) 26(8):2498–502. doi: 10.1093/ndt/gfq785 21303967

[15] Taki K Nakamura S Miglinas M Enomoto A Niwa T. Accumulation of Indoxyl Sulfate in OAT1/3-Positive Tubular Cells in Kidneys of Patients With Chronic Renal Failure. J Ren Nutr (2006) 16(3):199–203. doi: 10.1053/j.jrn.2006.04.020 16825019

[16] Wang W Hao G Pan Y Ma S Yang T Shi P. Serum Indoxyl Sulfate is Associated With Mortality in Hospital-Acquired Acute Kidney Injury: A Prospective Cohort Study. BMC Nephrol (2019) 20(1):57. doi: 10.1186/s12882-019-1238-9 30764800 PMC6376694

[17] Dou L Poitevin S Sallee M Addi T Gondouin B McKay N. Aryl Hydrocarbon Receptor is Activated in Patients and Mice With Chronic Kidney Disease. Kidney Int (2018) 93(4):986–99. doi: 10.1016/j.kint.2017.11.010 29395338

[18] Shi K Wang F Jiang H Liu H Wei M Wang Z. Gut Bacterial Translocation may Aggravate Microinflammation in Hemodialysis Patients. Dig Dis Sci (2014) 59(9):2109–17. doi: 10.1007/s10620-014-3202-7 24828917

[19] Lameire N. Nephrotoxicity of Recent Anti-Cancer Agents. Clin Kidney J (2014) 7(1):11–22. doi: 10.1093/ckj/sft135 25859345 PMC4389154

[20] Izzedine H Perazella MA. Anticancer Drug-Induced Acute Kidney Injury. Kidney Int Rep (2017) 2(4):504–14. doi: 10.1016/j.ekir.2017.02.008 PMC5720534 29318217

[21] Ozkok A Edelstein CL. Pathophysiology of Cisplatin-Induced Acute Kidney Injury. BioMed Res Int (2014) 2014:967826. doi: 10.1155/2014/967826 25165721 PMC4140112

[22] Chambers LM Kuznicki M Yao M Chichura A Gruner M Reizes O. Impact of Antibiotic Treatment During Platinum Chemotherapy on Survival and Recurrence in Women With Advanced Epithelial Ovarian Cancer. Gynecol Oncol (2020) 159(3):699–705. doi: 10.1016/j.ygyno.2020.09.010 32950250

[23] Famulski KS de Freitas DG Kreepala C Chang J Sellares J Sis B. Molecular Phenotypes of Acute Kidney Injury in Kidney Transplants. J Am Soc Nephrol (2012) 23(5):948–58. doi: 10.1681/ASN.2011090887 PMC3338297 22343120

[24] Nakagawa S Nishihara K Miyata H Shinke H Tomita E Kajiwara M. Molecular Markers of Tubulointerstitial Fibrosis and Tubular Cell Damage in Patients With Chronic Kidney Disease. PloS One (2015) 10(8):e0136994. doi: 10.1371/journal.pone.0136994 26317775 PMC4552842

[25] Qualls JE Kaplan AM van Rooijen N Cohen DA. Suppression of Experimental Colitis by Intestinal Mononuclear Phagocytes. J Leukoc Biol (2006) 80(4):802–15. doi: 10.1189/jlb.1205734 16888083

[26] Bolger AM Lohse M Usadel B. Trimmomatic: A Flexible Trimmer for Illumina Sequence Data. Bioinformatics (2014) 30(15):2114–20. doi: 10.1093/bioinformatics/btu170 PMC4103590 24695404

[27] Bolyen E Rideout JR Dillon MR Bokulich NA Abnet CC Al-Ghalith GA. Reproducible, Interactive, Scalable and Extensible Microbiome Data Science Using QIIME 2. Nat Biotechnol (2019) 37(8):852–7. doi: 10.1038/s41587-019-0209-9 PMC7015180 31341288

[28] Callahan BJ McMurdie PJ Rosen MJ Han AW Johnson AJA Holmes SP. DADA2: High-Resolution Sample Inference From Illumina Amplicon Data. Nat Methods (2016) 13(7):581. doi: 10.1038/nmeth.3869 27214047 PMC4927377

[29] Quast C Pruesse E Yilmaz P Gerken J Schweer T Yarza P. The SILVA Ribosomal RNA Gene Database Project: Improved Data Processing and Web-Based Tools. Nucleic Acids Res (2012) 41(D1):D590–D6. doi: 10.1093/nar/gks1219 PMC3531112 23193283

[30] Katoh K Misawa K Kuma Ki Miyata T. MAFFT: A Novel Method for Rapid Multiple Sequence Alignment Based on Fast Fourier Transform. Nucleic Acids Res (2002) 30(14):3059–66. doi: 10.1093/nar/gkf436 PMC135756 12136088

[31] Lane D. 16s/23s rRNA Sequencing. In: Nucleic Acid Techniques in Bacterial Systematics. New York: John Wiley and Sons (1991). p. 115–75.

[32] Price MN Dehal PS Arkin AP. FastTree 2–Approximately Maximum-Likelihood Trees for Large Alignments. PloS One (2010) 5(3):e9490. doi: 10.1371/journal.pone.0009490 20224823 PMC2835736

[33] Stecher G Tamura K Kumar S. Molecular Evolutionary Genetics Analysis (MEGA) for macOS. Mol Biol Evol (2020) 37(4):1237–9. doi: 10.1093/molbev/msz312 PMC7086165 31904846

[34] Mishima E Fukuda S Mukawa C Yuri A Kanemitsu Y Matsumoto Y. Evaluation of the Impact of Gut Microbiota on Uremic Solute Accumulation by a CE-TOFMS-Based Metabolomics Approach. Kidney Int (2017) 92(3):634–45. doi: 10.1016/j.kint.2017.02.011 28396122

[35] Wikoff WR Anfora AT Liu J Schultz PG Lesley SA Peters EC. Metabolomics Analysis Reveals Large Effects of Gut Microflora on Mammalian Blood Metabolites. Proc Natl Acad Sci USA (2009) 106(10):3698–703. doi: 10.1073/pnas.0812874106 PMC2656143 19234110

[36] Peng YS Lin YT Chen Y Hung KY Wang SM. Effects of Indoxyl Sulfate on Adherens Junctions of Endothelial Cells and the Underlying Signaling Mechanism. J Cell Biochem (2012) 113(3):1034–43. doi: 10.1002/jcb.23435 22213462

[37] Vaziri ND Yuan J Norris K. Role of Urea in Intestinal Barrier Dysfunction and Disruption of Epithelial Tight Junction in Chronic Kidney Disease. Am J Nephrol (2013) 37(1):1–6. doi: 10.1159/000345969 23258127 PMC3686571

[38] Sun CY Hsu HH Wu MS. P-Cresol Sulfate and Indoxyl Sulfate Induce Similar Cellular Inflammatory Gene Expressions in Cultured Proximal Renal Tubular Cells. Nephrol Dial Transplant (2013) 28(1):70–8. doi: 10.1093/ndt/gfs133 22610984

[39] Sun CY Hsu HH Wu MS. P-Cresol Sulfate and Indoxyl Sulfate Induce Similar Cellular Inflammatory Gene Expressions in Cultured Proximal Renal Tubular Cells. Nephrol Dial Transplant (1093) 28(1):70–8. doi: 10.1093/ndt/gfs133 22610984

[40] Hu J Zhong X Yan J Zhou D Qin D Xiao X. High-Throughput Sequencing Analysis of Intestinal Flora Changes in ESRD and CKD Patients. BMC Nephrol (2020) 21(1):12. doi: 10.1186/s12882-019-1668-4 31931722 PMC6958730

[41] Lau WL Savoj J Nakata MB Vaziri ND. Altered Microbiome in Chronic Kidney Disease: Systemic Effects of Gut-Derived Uremic Toxins. Clin Sci (Lond) (2018) 132(5):509–22. doi: 10.1042/CS20171107 29523750

[42] Devlin AS Marcobal A Dodd D Nayfach S Plummer N Meyer T. Modulation of a Circulating Uremic Solute *via* Rational Genetic Manipulation of the Gut Microbiota. Cell Host Microbe (2016) 20(6):709–15. doi: 10.1016/j.chom.2016.10.021 PMC5159218 27916477

[43] Yang JY Lee YS Kim Y Lee SH Ryu S Fukuda S. Gut Commensal Bacteroides Acidifaciens Prevents Obesity and Improves Insulin Sensitivity in Mice. Mucosal Immunol (2017) 10(1):104–16. doi: 10.1038/mi.2016.42 27118489

[44] Yanagibashi T Hosono A Oyama A Tsuda M Suzuki A Hachimura S. IgA Production in the Large Intestine is Modulated by a Different Mechanism Than in the Small Intestine: Bacteroides Acidifaciens Promotes IgA Production in the Large Intestine by Inducing Germinal Center Formation and Increasing the Number of IgA+ B Cells. Immunobiology (2013) 218(4):645–51. doi: 10.1016/j.imbio.2012.07.033 22940255

[45] Jacobson A Lam L Rajendram M Tamburini F Honeycutt J Pham T. A Gut Commensal-Produced Metabolite Mediates Colonization Resistance to Salmonella Infection. Cell Host Microbe (2018) 24(2):296–307.e7. doi: 10.1016/j.chom.2018.07.002 30057174 PMC6223613

[46] Then CK Paillas S Wang X Hampson A Kiltie AE. Association of Bacteroides Acidifaciens Relative Abundance With High-Fibre Diet-Associated Radiosensitisation. BMC Biol (2020) 18(1):102. doi: 10.1186/s12915-020-00836-x 32811478 PMC7437060

[47] Nakajima A Sasaki T Itoh K Kitahara T Takema Y Hiramatsu K. A Soluble Fiber Diet Increases Bacteroides Fragilis Group Abundance and Immunoglobulin A Production in the Gut. Appl Environ Microbiol (2020) 86(13):e00405–20. doi: 10.1128/AEM.00405-20 PMC7301863 32332136

