# Supplementary material for: Antibiotic Exposure Aggravates Bacteroides-Linked Uremic Toxicity in the Gut-Kidney Axis
Source: Front Immunol. 2022 Mar 24;13:737536. doi: 10.3389/fimmu.2022.737536 (PMC8988921; doi:10.3389/fimmu.2022.737536)
Supplement: Supplementary file 1 [file Image_1.pdf]

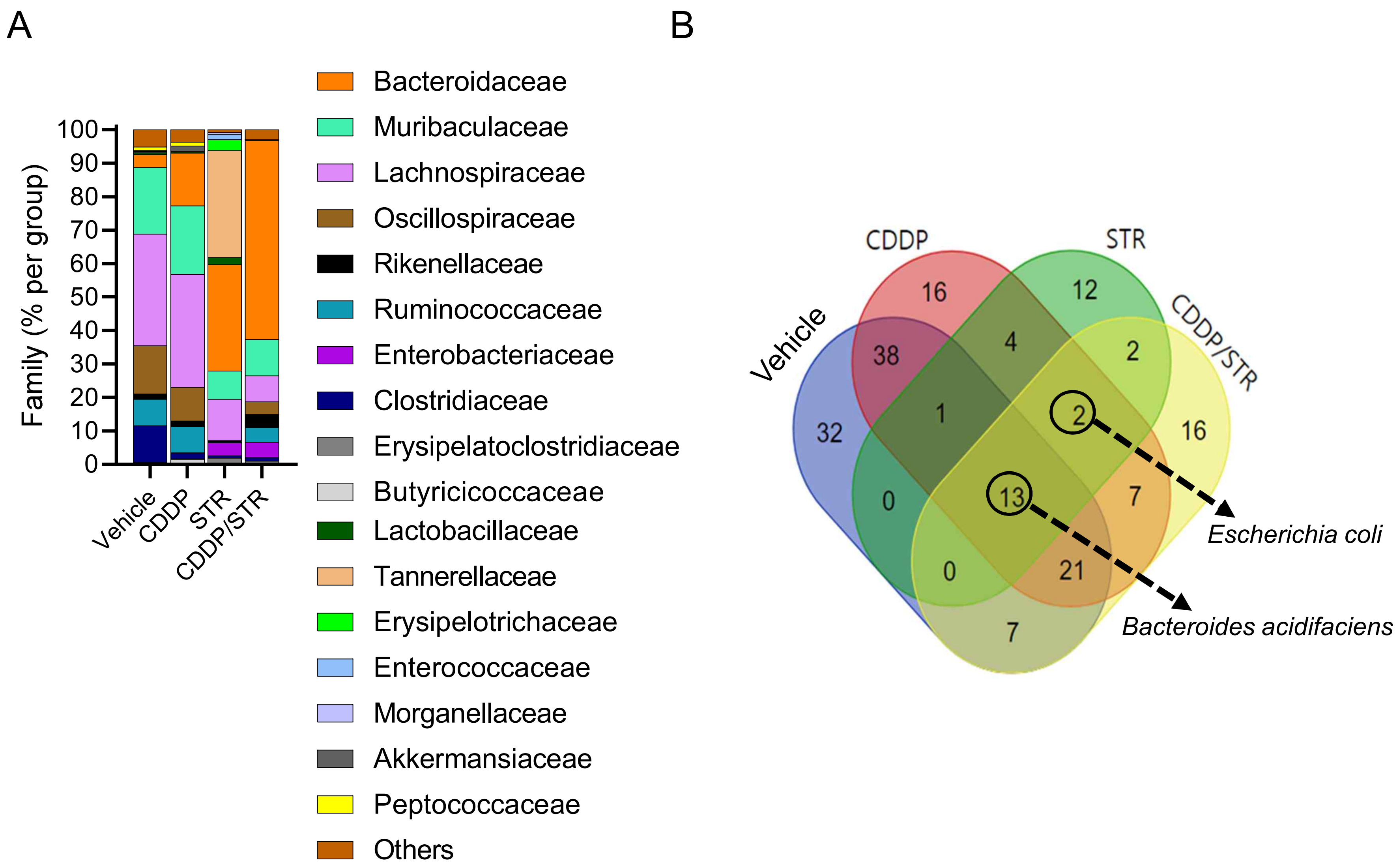

Supplementary figure 1. Effects of STR exposure on the murine gut microbiota profile in CDDP-induced renal injury model. Eight-week-old wild type male mice (n = 3–5) were orally preexposed to the vehicle or streptomycin (20 mg/mouse) for 24 h and then renal injury was induced with CDDP (20 mg/kg) via intraperitoneal administration for an additional 72 h. **(A)** The fecal bacteria were subjected to 16S rRNA analysis for phylogenetic composition at the family level (the mean relative abundances of families). **(B)** Grouping of bacterial community in the experimental mice based on the ontological clustering.
